# Hot Deformation Behavior and Simulation of Hot-Rolled Damage Process for Fine-Grained Pure Tungsten at Elevated Temperatures

**DOI:** 10.3390/ma15228246

**Published:** 2022-11-20

**Authors:** Yongqi Lv, Siqi Zhao, Tao Liu, Huichao Cheng, Jinglian Fan, Yuanchun Huang

**Affiliations:** 1 State Key Laboratory of Powder Metallurgy, Central South University, Changsha 410083, China; 2 Research Institute of Light Alloy, Central South University, Changsha 410083, China

**Keywords:** fine-grained pure tungsten, hot deformation behavior, constitutive equation, finite element model (FEM), hot rolling process, crack damage

## Abstract

Fine-grained pure tungsten fabricated by a sol drying reduction low-temperature sintering method and hot isothermal compression tests were performed by using the Gleeble 3800 thermo mechanical simulator at deformation temperatures from 1273 K to 1473 K and strain rates from 0.001 s^−1^ to 1 s^−1^. In addition, the constitutive equation was established by least square method combined with the Zerilli–Armstrong model, and the hot deformation behavior was discussed. Moreover, based on constitutive equation, the influence of the rolling process and its parameters on temperature, strain, density and rolling force in the hot rolling process was investigated at elevated temperature by the finite element model (FEM). Furthermore, the form of rolling damage and its formation mechanism were analyzed. Results showed the grains of pure tungsten are dense, irregular polyhedral spherical and very fine, and the average grain size is about 5.22 μm. At a high strain rate, the flow stress increases rapidly with the increase in strain, while the stress–strain curve shows a flattening trend in the tested strain rate range with increasing temperature, and no flow stress peak exists, showing obvious dynamic recovery characteristics. Furthermore, the FEM simulation showed that compared with the rolling temperature, the reduction has a greater influence on the temperature, stress–strain field and its distribution. There are three kinds of damage in the hot rolling process: transverse cracks, longitudinal cracks and side cracks, which are attributed to the competition between additional stress caused by uneven deformation and material strength. Moreover, the control method of hot rolling defects had been preliminarily proposed. These results should be of relevance for the optimum design of the hot rolling process of pure tungsten.

## 1. Introduction

Tungsten, as a high temperature structure material, is widely used in aerospace, the military and electronic industries as well as other cutting-edge fields because of its unique set of properties, such as high melting point, high elastic modulus, high thermal conductivity and excellent mechanical properties at elevated temperatures [1,2,3,4]. Simultaneously, in recent decades, tungsten has been determined as plasma-facing material in fusion reactors due to its low sputtering yield, high irradiation properties, and good plasma compatibility, which has attracted great attention from researchers at home and abroad [5,6,7,8,9]. However, currently, pure tungsten has some disadvantages, such as coarse grain size, high recrystallization temperature (<1200 °C) and low ductile–brittle transition temperature (DBTT, >400 °C) [10,11,12]. Particularly, the brittleness at room temperature caused by high DBTT is the major shortcoming of tungsten materials [13], resulting in serious challenges for its workability and performance, which seriously hinders demanding applications of tungsten materials in advanced and sophisticated fields. Reducing DBTT and improving room ductility of tungsten materials have become the key problems, which could be solved urgently.

For the past several decades, an important way to improve the ductility of tungsten is to add alloying elements [14,15], such as rhenium, titanium, tantalum, zirconium and molybdenum [16,17,18,19,20]. Hatanoa et al. [21] reported that adding rhenium can reduce DBTT and improve ductility of tungsten materials, in particular, W-26%Re alloys exhibit plasticity at room temperature [22], but rhenium is too expensive. To date, the effect of other alloying elements on the room temperature ductility of tungsten materials is not clear. However, in contrast, research by Tejado et al. [23] reported that the non-uniform dispersion of Ti may result in higher DBTT of W-Ti alloy than that of pure tungsten. Recently, researchers pointed out that adding nano-ceramics, such as TiC, ZrC, La_2_O_3_ and Y_2_O_3_ [24,25,26,27], to prepare nanocrystalline or fine-grained structures can improve the toughness and the ductility of tungsten. Though, significant progress has been made on improving the toughness of tungsten materials by additions of alloying elements and nano-ceramics, there is no strong evidence that tungsten is malleable in the as-sintered state. Simultaneously, the complexity of composition and structure affects the material preparation and recyclability [28,29]. In contrast, deformation processing has the most effective method to improve the ductility and decrease DBTT of tungsten materials [30,31,32,33]. Shen et al. reported that the DBTT of as-rolled tungsten at 1450 °C can be reduced from above 700 °C to less than 300 °C [34]. Similar results were observed by Levin et al. using room temperature tensile testing [35]. The elongation of pure tungsten with equal channel angular extrusion (ECAE) is 17–23%. However, it is known that the brittleness and recrystallization temperature of pure tungsten are relatively high, and the hot working process is usually performed at high temperature (>1200 °C), which will lead to high energy consumption and high cost in the thermal working test of pure tungsten. Moreover, compared with the experimental study, the numerical simulation has the unique advantages of simplicity, convenience and high efficiency; therefore, it becomes an effective way to study the thermal processing and deformation characteristics of pure tungsten. Nevertheless, there are few studies on the simulation of hot working for pure tungsten, and only the coarse particles of pure tungsten have been studied [36]. Compared with ordinary pure tungsten, fine-grained pure tungsten has higher performance and greater development potential. Unfortunately, the hot deformation behavior, hot rolling process and damage behavior of fine-grained pure tungsten have not been studied.

In this work, the hot deformation behavior of fine-grained pure tungsten was studied by hot compression tests at elevated temperature. In addition, using the true stress–strain data obtained by experiments, the constitutive equations of fine-grained pure tungsten was established. Moreover, based on the constitutive equations, the process and damage behavior during hot rolling were analyzed through the finite element simulation by using Deform-3D software, and the damage mechanism was also preliminarily discussed, which can provide theoretical data for the hot rolling test of pure tungsten.

## 2. Experimental Procedures

The nano powders of pure tungsten as the raw material were fabricated by the nano in situ composites method, and the detailed process and powder characteristics can be referred to our previous study [37]. Then, the pure tungsten slab with 150 × 100 mm^2^ and thickness of 20 mm was obtained by cold isostatic pressing and ordinary sintering in H_2_ atmosphere at 1850–1950 °C. The microstructure of sintered samples was characterized by scanning electron microscopy (SEM, JSM-6360LV, JEOL, Tokyo, Japan).

The hot compression specimens were machined to cylinders with dimension of ϕ8 × 12 mm. The hot compression tests was performed with a Gleeble 3800 simulator within a temperature range of 1273–1473 K and a strain rate region of 0.01–1 s^−1^ up to a total strain of 0.6.

Moreover, the temperature, strain, density, roll force and its distribution under the hot rolling process were predicted and analyzed by finite element modelling (FEM) using Deform-3D software [38,39]. The cuboid tested sample with the size of 150 mm × 100 mm × 20 mm was selected as the object structure for the analysis. In order to reduce the computational burden, the cuboid tests were modeled by a quarter solid element because the stress is symmetric in the rolling process. Moreover, the cuboid sample was meshed with the free mesh method. The material model of fine-grained pure tungsten does not exist in the Deform-3D software. Therefore, except for Young’s modulus and Poisson’s ratio, which refer to relevant papers [40,41], the material density, liner expansion coefficient and specific heat capacity were measured experimentally. Based on the above experiments, the material data and model of fine-grained pure tungsten were reconstructed in Deform-3D software. Material parameters of samples used in FEM are listed in Table 1. In this FEM, boundary conditions were defined in accordance with the experimental hot rolling process. The position of the roller was determined according to the designed amount of pressure, and the friction between the roller and the tungsten plate was selected as the shear friction. Additionally, in order to ensure the bite of the tungsten plate during the simulation, a fixed speed push block with 0.333 m/s (linear velocity) was set to provide thrust. Considering the inertia effect, the kinetic algorithm was used to better calculate the fast rolling process. For facilitating the presentation, the rolling direction, the transverse direction of the rolled samples and the normal direction of the rolled surface were marked as RD, TD and ND, respectively, which correspond to the X, Y and Z axes. Moreover, to facilitate the finite element simulation, based on the following assumptions: (1) there is just heat conduction and no surface reflection for the convenience simulation, (2) material parameters remain constant, (3) rolling along the direction is perpendicular to the paper facing out, and is unidirectional.

## 3. Results and Discussion

### 3.1. Constitutive Models for Fine-Grained Pure Tungsten at Elevated Temperatures

**Figure 1:**
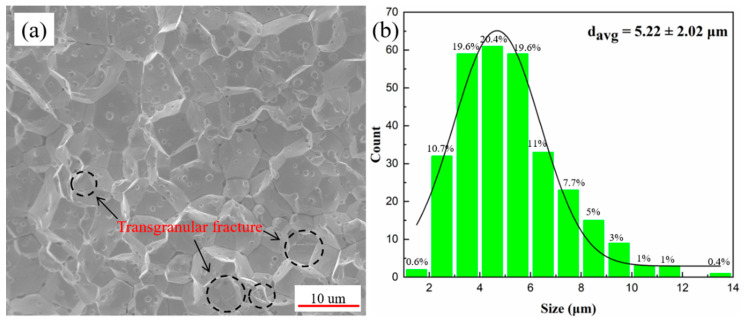
SEM image of fractured surfaces (**a**), and the grain size distributions (**b**) of sintered pure W.

Figure 1 shows the SEM image of fractured surfaces (a), and the grain size distributions (b) of the as-sintered fine-grained pure tungsten. As can be seen from Figure 1a, the grains of pure tungsten were polyhedral spherical and arranged compactly. In addition, the fracture structure was whole and smooth, showing typical intergranular fracture characteristics. However, there is a small amount of transgranular fracture, as shown by the black dotted circles in Figure 1a. The original sintered pure tungsten shows a fine grain size and uniform distribution. The average grain size and its distribution of pure W were determined from 300 grains in different regions of SEM images [42], as shown in Figure 1b. The grain size is concentrated in 2.3–7.5 μm, and the number of grains in this range accounts for about 82% of the total statistical grain size. Moreover, the average grain size of pure W is about 5.22 μm, showing a significant refinement effect.

**Figure 2:**
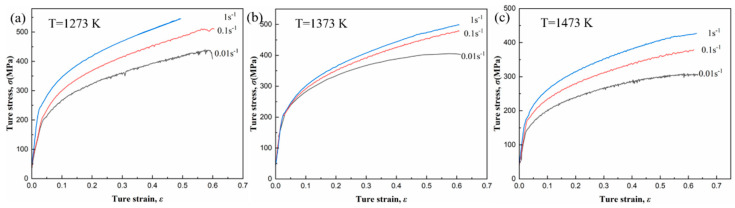
True stress−strain curves of fine-grained pure tungsten obtained from hot compression tests at different strain rates with various temperatures: (**a**) 1273 K, (**b**) 1373 K, (**c**) 1473 K.

Typical hot compressive true stress–strain curves of fine-grained pure tungsten with strain rates of 0.01, 0.1 and 1 s^−1^ from 1273 K to 1473 K are shown in Figure 2. It can be seen the flow stress increases rapidly with the increase in strain at the initial stage of hot deformation. This is because the formation of new dislocations at the initial stage of hot compression leads to the increase in dislocation density and the significant work hardening [43,44]. However, with the further increase in strain (*ε* > 0.6), the increasing trend of flow stress becomes slow which are attributed to thermal activation softening caused by dislocation rearrangement and annihilation during the compression process [45]. Moreover, the flow stress always showed an increasing trend with the increase in strain, and no obvious peak stress appears when the hot compression temperature is 1273 K as shown in Figure 2a. A similar phenomenon was also observed at hot compression temperatures up to 1373 and 1473 K as shown in Figure 2b,c. Nevertheless, the increasing trend of flow stress becomes gentle, especially, the stress curves tend to a steady stage at low strain rates (0.01 s^−1^). Therefore, the stress–strain curve can be divided into three stages: (1) the micro-strain stage, where the flow stress increases rapidly under a very small strain and the deformation leads to the rise in dislocation density, which results in work hardening; (2) the uniform deformation stage, where the flow stress continues to increase with the increase in strain; however, the slope of the stress–strain curve gradually decreases, and the softening effect offends part of the work hardening effect; (3) the steady flow stage, where the flow stress no longer increases with the increase in strain and remains in a horizontal state, and there is a constant stress. In this case, the softening effect completely cancels the work hardening caused by deformation, i.e., the rate of increase in dislocation density and that of annihilation of dislocations reaches equilibrium [46]. Thus, the dynamic equilibrium of work softening and work hardening is obtained [47]. The micro-strain stage and the uniform deformation stage were usually observed under all circumstances as shown in Figure 2a–c; however, the steady flow stage was most obviously observed only when the deformation temperature is high and strain rate is low (T = 1473 K and $\dot{\varepsilon }$ = 0.01 s^−1^), as shown in Figure 2c, which is due to the small range of temperature and strain rate selected in the hot compression test. Nevertheless, in relevant studies [48,49], the true stress strain curve usually shows a partial drop area, wherein there is a continuous decrease in flow stress with the increase in deformation temperature. In addition, a stress peak is observed, which is related to dynamic recrystallization (DRX). Moreover, no peak of flow stress was formed in the stress–strain curve of this work, which is related to dynamic recovery (DRV) [50]. The dynamic recovery will increase with the increase in applied strain, and lead to the enhancement of softening effect. But softening rate caused by DRV is lower than work hardening rate which leads to the continuous increase in flow stress. As the strain continues to increase, the softening effect induced by dynamic recovery increases until it is balanced with work hardening. Based on the characteristics of the true stress–strain curves above, commonly, the hot deformation of fine-grained pure tungsten exhibits typical dynamic recovery (DRV) characteristics.

Moreover, further analysis of Figure 2 shows that, for a constant deformation temperature, the flow stress increases with the increase in strain rate when the strain rate is high (0.1 and 1 s^−1^). A similar trend is followed by the pure tungsten at other temperatures 1273, 1373 and 1473 K. When the temperature is 1273 K, the flow stress with strain rate of 0.01 s^−1^ shows the same phenomenon. While the temperature is 1373 and 1473 K, the stress curves remain flat at low strain rate of 0.01 s^−1^. At elevated temperatures and low strain rate, the increase in thermal activation for atoms and the acceleration of dislocation movement can result in the balance between softening and work hardening. Simultaneously, for a particular strain rate, the flow stress decreases with the rise in deformation temperature, which can be attributed to the fact that, at lower temperatures, the amount of work hardening is compensated by flow softening. Therefore, the stress–strain curve characteristics of fine-grained pure tungsten during hot compression are largely the result of dynamic competition between work hardening and thermal activation softening. This indicates that temperature and strain rate are the governing factors, which play the key roles in the hot deformation behavior of fine-grained pure tungsten. Unfortunately, the characterization and analysis of deformed microstructures have not yet been performed, which will require further discussion and research.

The constitutive model is an important way to accurately describe the flow stress–strain relation of metallic materials. Commonly used models are the Arrhenius model, Johnson–Cook (JC) model, Khan–Huang–Liang (KHL) model, Zerilli–Armstrong (ZA) model and MTS model. According to the reports in the literatures [36,51,52], the ZA model is more suitable for the establishment of the constitutive equation of fine-grained tungsten materials because it is proposed based on the thermal activation theory of dislocation which comprehensively considers the effects of strain hardening, strain rate hardening and thermal softening. Simultaneously, it has a simpler form than the other constitutive models based on dislocation theory. Moreover, in Deform software, the ZA model is one of the models that can be directly called, which is convenient to apply. Moreover, this model also fully reflects the effect of grain size. Therefore, the ZA model is selected to construct the constitutive equation of fine-grained pure tungsten. For materials with different crystalline forms (FCC, BCC and HCP), the ZA model has different expressions. In this paper, the ZA model was selected, and the flow stress of fine-grained pure tungsten BCC structure can be expressed as

(1) $$\sigma =\sigma_{0}+c_{1}exp\left(-c_{3}T+c_{4}T\ln\dot{\varepsilon }\right)+c_{5}\varepsilon^{n}$$

where, *T* is the deformation temperature, *ε* is strain and $\dot{\varepsilon }$ is strain rate. c_1_ is an interior variable changing with dislocation density motion. Moreover, *σ*_0_ is the non-thermal part of the yield stress, which is related to the grain size of the material. The *σ*_0_ of Equation (1) can be obtained by the Hall–Petch hardening function, which can be expressed as

(2) $$\sigma_{0}=\Delta \dot{\sigma_{G}}+Kd^{-0.5}$$

where, $\Delta \dot{\sigma_{G}}$ is the additional part of stress, *K* is the microscopic stress intensity and *d* is the average grain size.

Equation (1) was solved and numerically fitted by the least square method. The least square method can be expressed by Equation (3):

(3) $$\mathrm{Min}\left[\sum {\left(Y_{i}-Y_{j}\right)}^{2}\right]$$

where, Min is the instruction to use the minimum value, *Y_i_* and *Y_j_* are the experimentally measured value and the calculated value with unknown parameters of the function.

For the ZA constitutive equation of fine-grained pure tungsten, there are many variables to be considered in the fitting process, for example, *T*, *ε* and $\dot{\varepsilon }$ are the independent variables of σ. The calculation is complicated, so Equation (1) was adjusted to the software instruction of MATTHEMATICA, which can be expressed as Equation (4):

(4) $$\mathrm{Minimize}\{[\mathrm{Sum}[\mathrm{Sum}\left[\mathrm{Sum}{\left[Y_{i}-Y_{j}\right]}^{2},\left\{i,1,3\right\}\right],\left\{j,1,3\right\}],\left\{k,1,m\right\}],Y_{i}>0\&Y_{j}>0\}$$

Here, Y stands for σ in Equation (1). Substituting Equation (1) into Equation (4) leads to the following expression:

(5) $$\begin{array}{c} \mathrm{Minimize}\{[\mathrm{Sum}[\mathrm{Sum}\left[\mathrm{Sum}{\left[\sigma_{0}+c_{1}\exp\left(-c_{3}T_{i}+c_{4}T_{i}\ln{\dot{\varepsilon }}_{j}\right)+c_{5}\varepsilon_{k}{}^{n}\right]}^{2},\left\{i,1,3\right\}\right],\left\{j,1,3\right\}],\left\{k,1,m\right\}],\\ \sigma_{0}>0\&\&c_{1}>0\&\&c_{3}>0\&\&c_{4}>0\&\&c_{5}>0\&\&n>0],[\sigma_{0},c_{1},c_{3},c_{4},c_{5}]\} \end{array}$$

where, *T_i_* is the *i*th value of temperature, and ${\dot{\varepsilon }}_{j}$ is the *j* th value of strain rate. Simultaneously, $\varepsilon_{k}$ is the *k*th value of strain. That is, *T*_1_, ${\dot{\varepsilon }}_{1}$ and $\varepsilon_{1}$ are the first values of temperature, strain rate and strain, respectively.

Based on the true stress–strain curves show in Figure 2, the test data were substituted into Equation (5) to fit the equation parameters, and the fitting results are listed in Table 2. Substituting the fitting parameters of Table 2 into Equation (1), the constitutive equation based on ZA model can be obtained:

(6) $$\sigma =121.4+187.0exp\left(-0.094T+0.1T\ln\dot{\varepsilon }\right)+418.59\varepsilon^{0.477}$$

### 3.2. Effect of Starting Rolling Temperature and Rolling Reduction on Rolling Behavior

As key process parameters, the rolling starting temperature and the rolling reduction have great influence on rolling microstructure and properties. Through the finite element simulation using Deform-3D software, the influencing regularity of the rolling starting temperature and the rolling reduction on the temperature field, stress–strain field and its distribution during hot rolling process has been analyzed and discussed.

**Figure 3:**
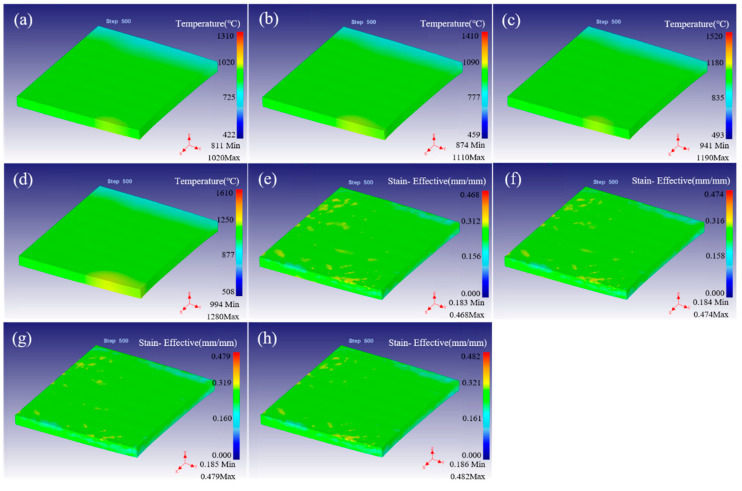
The distribution of temperature field (**a**–**d**), and strain field (**e**–**h**) by FEM simulation under different rolling temperatures of 1300–1600 °C.

The distribution of temperature and strain for fine-grained tungsten at different rolling temperature with reduction of 20% were simulated, as shown in Figure 3. The distribution of temperature field is basically the same and the distribution is relatively uniform at different temperatures, as shown in Figure 3a–d. The temperature at the inlet end is higher and the temperature at the tail end is lower, which is due to the inconsistent temperature drop at the front and back end caused by heat conduction during rolling. Moreover, under the hot rolling temperature of 1300 °C, the highest temperature of the surface in the as-rolled region is 1020 °C, while the rolling temperature is 1400 °C and the highest surface temperature is 1100 °C. When the rolling temperature increases to 1500 °C and 1600 °C, the maximum surface temperature is 1190 °C and 1280 °C, respectively. This indicates that the surface temperature increases with the increase in rolling temperature, but the magnitude of the change is smaller. Simultaneously, as the rolling temperature increases, the drop in surface temperature increases. This is because the higher the temperature, the larger the temperature gradient in the same environment. It makes it easy to have thermal radiation/conduction leading to a large drop in temperature. Moreover, there is a region with high local temperature at the edge at the entrance end of rolling. This strange phenomenon may be one of the reasons for the cracking damage in the hot rolling process, which is studied in detail in the next part.

Figure 3e–h showed strain distribution at different rolling temperatures. When the rolling temperature is between 1300–1600 °C, the maximum strain is 0.468, 0.474, 0.479 and 0.482, respectively. The possible reason is that the rolling temperature increases, the material softens obviously, and the deformation is easier with the same reduction. Moreover, the strain distribution of the rolled samples is basically the same, which shows that the rolling temperature has little effect on the strain and its distribution when the rolling reduction is constant. The reduction is 20%, under the same conditions of deformed samples and its characteristics, the deformation along the Y axis direction (the ND direction) is the same, which means that the strain is the same. However, along the X axis (rolling direction), the higher the deformation temperature, the easier the deformation, resulting in the increase in the deformation amount. As there is no constraint around the rolling sample, a wide along rolling direction is formed in the process of self-adjustment inside the sample, which keeps the strain stable. The maximum strain is at the surface. Moreover, the strain of the upper and lower surfaces contacting the rolling rail is larger than that of the central region, which may be caused by the following two reasons: (1) high roll force on the surface in contact with the roll, (2) unconstrained or less constraints on the surface lead to deformation. Simultaneously, similar to the temperature field distribution, large local strain appears at the edge of the rolled sample at the inlet end, which is easy to cause uneven deformation and result in rolling damage.

**Figure 4:**
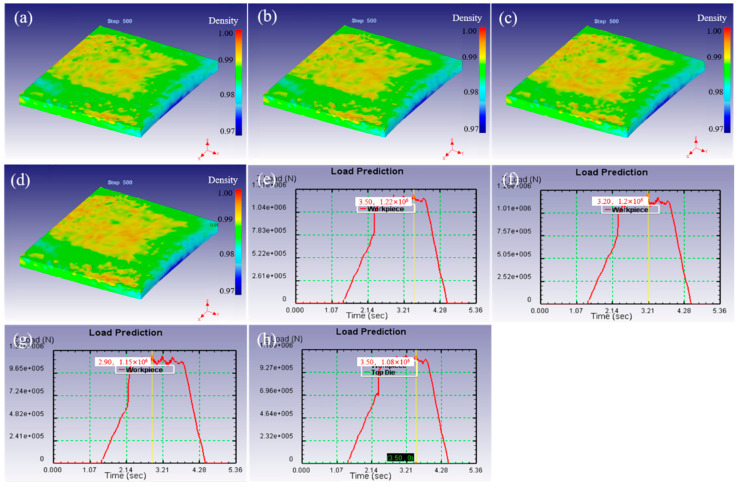
The distribution of density field (**a**–**d**), and roll force (**e**–**h**) by FEM simulation under different rolling temperatures of 1300–1600 °C.

Figure 4 shows the variation in density field distribution and rolling force under different rolling temperatures simulated by finite element method. The density and its distribution of as-rolled samples are roughly the same at different rolling temperatures. The density in the central area is high, and the density in the surrounding wide area is relatively low. This is because the constraining force in the surrounding wide area is small, and the rolling mainly leads to deformation expansion of the area around the slab rather than density increase, while the central part due to the difficulty of deformation expansion by constraining in all directions, rolling promotes the increase in density. Moreover, the density of the upper and lower surfaces of the as-rolled sample is higher than that of the inner surface for the same reason. This indicates that the rolling temperature has little effect on the density distribution when the reduction is constant. There is a situation of when the partial density is high in the front of the edge for the as-rolled sample, which is the same as the distribution of temperature and strain fields in Figure 3.

The rolling force of the as-rolled samples with a reduction of 20% at different rolling temperatures was analyzed, as shown in Figure 4e–h. The rolling temperature is 1300 °C, and the rolling force is 1.22 × 10^6^ N. When the temperatures increase to 1400, 1500 and 1600 °C, the rolling forces are 1.2 × 10^6^, 1.15 × 10^6^ and 1.08 × 10^6^ N, respectively. This indicates that the rolling force decreases with the increase in rolling temperature, which is beneficial to reduce the deformation resistance to a certain extent. The thermal activation process of fine tungsten is aggravated by the increase in rolling temperature, and the softening caused by dynamic recovery is enhanced. Simultaneously, with the increase in temperature, the atomic kinetic energy increases and the bonding force decrease, which leads to the decrease in the critical shear stress, forming a new slip system. Thus, the synergistic effect of the two causes the deformation resistance to decrease with the increase in temperature.

**Figure 5:**
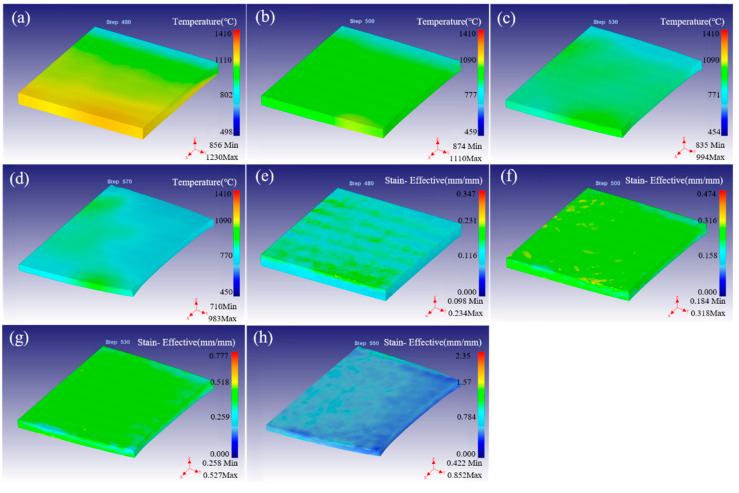
The distribution of temperature field (**a**–**d**), and strain field (**e**–**h**) by finite element simulation under different rolling reduction with 10–40%.

In order to study the influence of rolling reduction on the hot rolling process, simulations and analyses were carried out by selecting four kinds of pressing quantity with 1400 °C. Figure 5 shows the distribution of temperature field and strain field under different rolling reductions. When the amount of reduction is 10%, the highest surface temperature of the sample after rolling is 1230 °C, which is located in the front end of the bite. The lowest temperature is 856 °C, located at the bite end of the sample, as shown in Figure 5a. The surface temperature of the sample is in the range of 874–1110 °C when the rolling reduction is increased to 20%. While the rolling reductions are 30% and 40%, the surface temperatures are in the range of 835–994°C and 710–983 °C, respectively, as shown in Figure 5b–d. Similar to the temperature field distribution in Figure 3, the highest temperature of the sample appears on the surface of the bite front after rolling. Moreover, with the increase in the amount of reduction, the temperature of fine-grained W plate decreases while the temperature drop increases. In the case of a certain rolling speed, if the amount of reduction increases, the rolling time will increase and the greater temperature dropping will be caused. The strain field and its distribution of the rolled sample is very uneven at 10% of reduction, the maximum surface strain is 0.234 and the strain inside the plate is low as shown in Figure 5e. The uniformity of the rolling strain field is improved when the reduction increases to 20%, and the maximum surface strain is 0.318. In particular, the strain field distribution of the rolled sample is uniform when the reduction is 30% and 40%, and the maximum strain on the surface is 0.527 and 1.058, respectively, as shown in Figure 5f–h. The strain is low just in the peripheral local extension area. This indicates the strain increases significantly when the reduction increases. Moreover, the strain is high on the surface and low in the inner and peripheral wide parts. In particular, the phenomenon of local maximum strain was observed in the center of the bite end, which is easy to cause non-uniform local strain and lead to crack formation.

**Figure 6:**
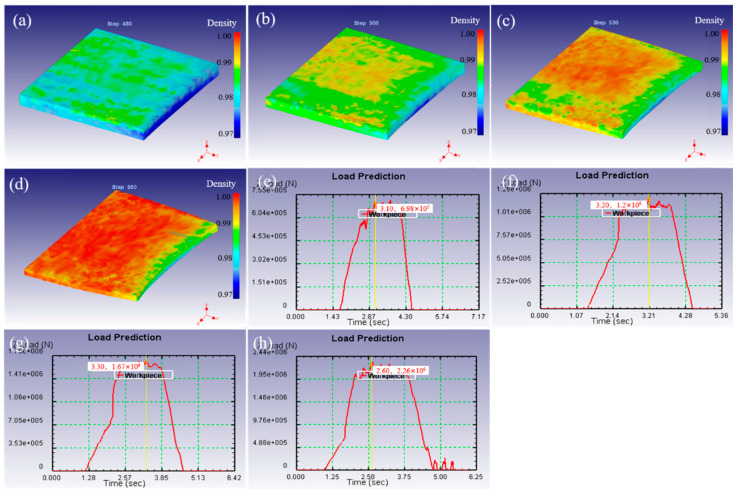
The distribution of density field (**a**–**d**), and roll force (**e**–**h**) by finite element simulation under different rolling reductions with 10–40%.

The density field distribution and rolling force under different rolling starting reduction were also simulated and analyzed, as shown in Figure 6. The surface density of the rolled sample is higher than that of the inner and peripheral wide-spread parts when the starting reduction is 10%, and according to the simulation results, the surface density is mainly between 0.973 and 0.980, as shown in Figure 6a. In particular, the local density reached above 0.983 in the middle of the bite end. Simultaneously, the density field and its distribution are very uneven, which is similar to the strain field in Figure 5. At the 20% point of starting the reduction, the density field of the rolled sample is basically unchanged, but the uniformity of the surface density field is improved. While the starting reduction is 30%, the surface density field distribution which becomes more uniform is mainly between 0.986 and 0.991. The amount of starting reduction continues to increase to 40%, a uniform density field on the surface of the sample is found, and most of the density reached 0.993, as shown in Figure 6b–d. The results indicates that the density of the rolled samples increases with the increase in the reduction, and the uniformity of the density field also shows a continuous increase. More importantly, the density of all the rolled samples was high on the surface, but low in the inner and surrounding widespread areas. Especially, locally high density area at the bite front was observed.

Moreover, further analysis of the effect of the amount of starting reduction on rolling force was carried out, as shown in Figure 6e–h. The simulated rolling forces are 6.98 × 10^5^, 1.2 × 10^6^, 1.67 × 10^6^ and 2.26 × 10^6^ N, respectively. This result shows the rolling force increases with the increase in i the amount of starting reduction. The possible reason is that the increase in starting reduction leads to the increase in deformation under a certain temperature, which results in an increase in the degree of work hardening. In order to ensure that the set amount of rolling reduction is achieved, it is necessary to provide a larger rolling force.

In a certain temperature range (1300–1600 °C), the effect of starting rolling temperature on the rolling process is not obvious. Nevertheless, the temperature field, strain field, density field, rolling force and their distribution are more sensitive to the starting rolling reduction. With the increase in the amount of reduction, the rolling surface temperature decreases, and the strain, density and rolling force increase significantly. More importantly, under low reduction, the distribution of temperature field, strain field and density field are extremely uneven, while under high reduction, their uniformities are greatly improved. However, the high reduction will lead to a great increase in rolling force, which is not conducive to hot rolling. Therefore, it is very meaningful to optimize the assign of rolling starting temperature and reduction, as to obtain high performance fine-grained W thin plate index.

### 3.3. Formation and Control of Rolling Cracks

Crack is the main damage form in the rolling process, which seriously affects the properties of rolled samples. However, the types and formation of cracks are mostly based on empirical analysis. Based on finite element simulation, the additional stress caused by the uneven deformation of special positions in rolled slabs under different rolling processes was calculated. Moreover, the distribution, types and formation mechanism of rolling cracks were preliminarily discussed and analyzed.

**Figure 7:**
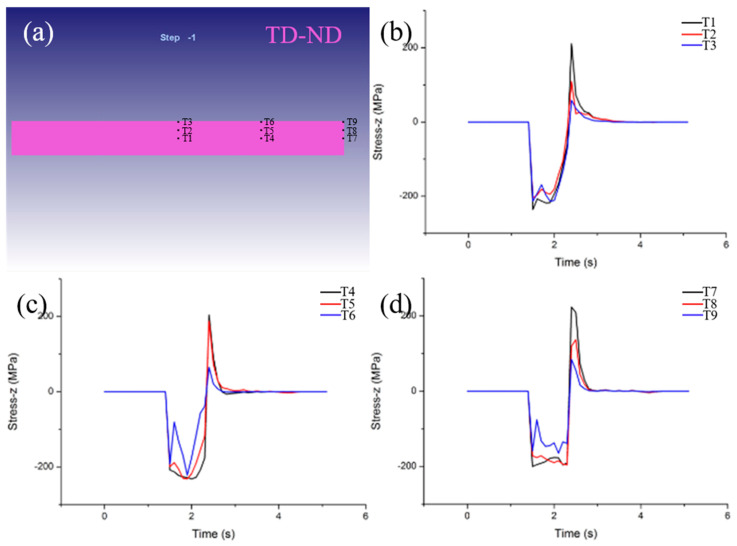
Distribution of characteristic points on TD−ND plane (**a**), the additive stress distribution along ND direction at set point on TD−ND plane at 1400 °C and 20% reduction (**b**–**d**).

Figure 7 showed the stress variation along the ND at 1400 °C and 20% reduction for selected positions on the TD−ND plane. During the hot rolling process, the additional tensile stress along the ND direction exists on the TD−ND plane, and the tensile stress at T1, T4 and T7 is the highest. In addition, the stress at (T1, T4, T7), (T2, T5, T8) and (T3, T6, T9) is basically similar. This indicates that the tensile stress is low on the surface and high on the inside along the thickness direction (ND). Meanwhile, the stress at the same depth along the width direction (TD) does not change. According to the results of Figure 3 and Figure 5 above, the strain on the rolled surface is large and in the inside is small, thus, the strain gradient is formed which induces the formation of the additive stress, and it increases continuously from the surface to the inside. Moreover, the competition between the additive tensile stress on the TD−ND plane and the yield strength of the rolled specimen can result in the formation of transverse cracks. Due to the unconstrained TD−ND plane of the bite end, the internal additive tensile stress is the largest, so the transverse crack is most likely to form on the TD−ND plane of the bite end.

**Figure 8:**
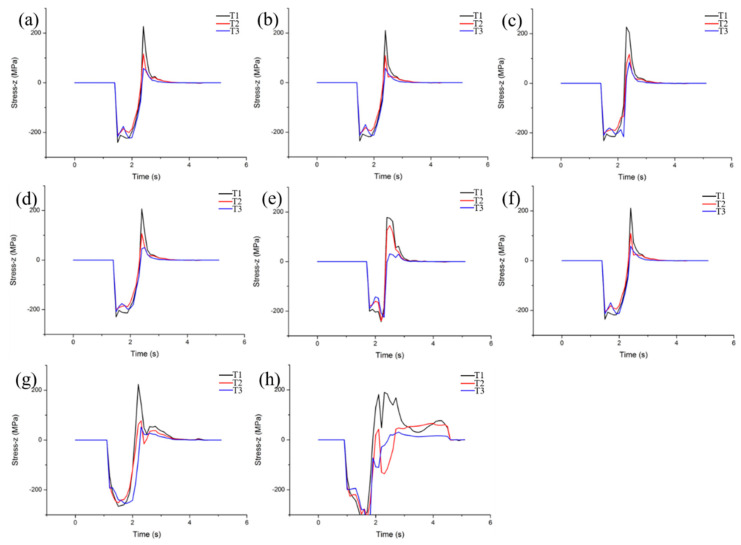
The additive stress distribution along ND direction at set point at different rolling temperatures and reduction: (**a**) 1300 °C, (**b**) 1400 °C, (**c**) 1500 °C, (**d**) 1600 °C, (**e**) 10%, (**f**) 20%, (**g**) 30% and (**h**) 40%.

In order to study the effect of the rolling process on transverse crack formation, three points of T1, T2 and T3 in TD−ND plane were selected to simulate and analyze additive tensile stress at different rolling temperatures and reduction, as shown in Figure 8. Figure 8a–d showed the stress distribution at different rolling temperatures with 20% reduction. The rolling temperature is 1300 °C, and the internal maximum stress at P1 is 227 MPa. When the temperature increases to 1400–1600 °C, the maximum tensile stress is 211 MPa, 209 and 207 MPa, respectively. This indicates that the critical additive tensile stress is not sensitive to the rolling temperature. Moreover, the critical stress of transverse cracks formed by the hot rolling of fine-grained tungsten plates is 227, 211, 209 and 207 MPa at 1300–1600 °C, respectively. When the yield strength of the material itself is greater than the above critical stress value, there will be no transverse crack damage, on the contrary, it will form transverse crack damage. The maximum additive stress on the TD−ND plane is 178, 211, 224 and 181 MPa when the rolling reduction is 10%, 20%, 30% and 40%, respectively, as shown in Figure 8e–h. This means that the reduction has a significant effect on the additive stress, and its value increases with the increase in the reduction. However, when the reduction increases to 40% and the strain is large enough, the uniformity of the overall strain of the rolled plate will be improved, so the maximum tensile stress decreases. Moreover, the critical stresses of transverse cracks formed by hot rolling of fine-grained tungsten plates are 178, 211, 224 and 181 MPa with reductions of 10–40%, respectively.

Therefore, the temperature can be chosen to be higher than 1400 °C, the amount of reduction is less than 20%, and the preparation process of rolled slab can be optimized to improve its high temperature strength, which can control and avoid the generation of transverse cracks.

**Figure 9:**
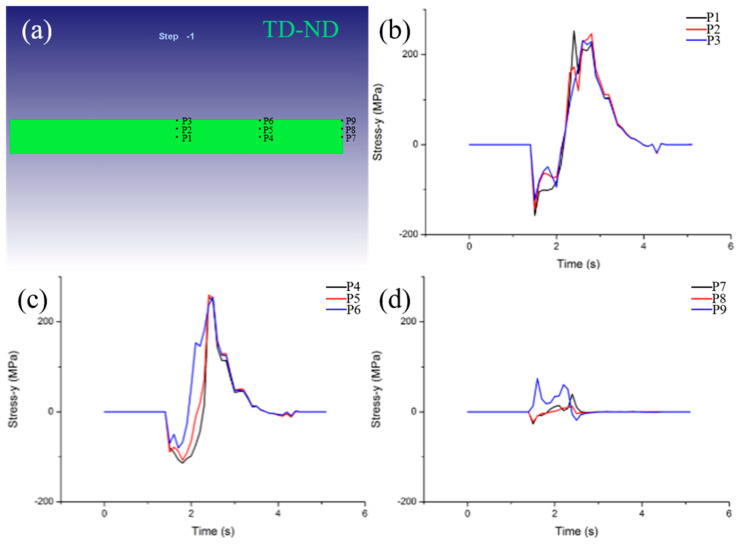
Distribution of characteristic points on TD−ND plane (**a**), the additive stress distribution along TD direction at set point on TD−ND plane at 1400 °C and 20% reduction (**b**–**d**).

The longitudinal crack is caused by the TD direction stress on the ND−TD plane during rolling, the tensile stress in TD direction at the selected position were simulated and analyzed, as shown in Figure 9. When the bite end of the rolled piece comes out of the roll gap, there is tensile stress in TD direction in the plate. In addition, the maximum value of the same position in different directions of thickness is not different, but the magnitude and distribution of tensile stress are significantly different at different widths along the TD direction.

**Figure 10:**
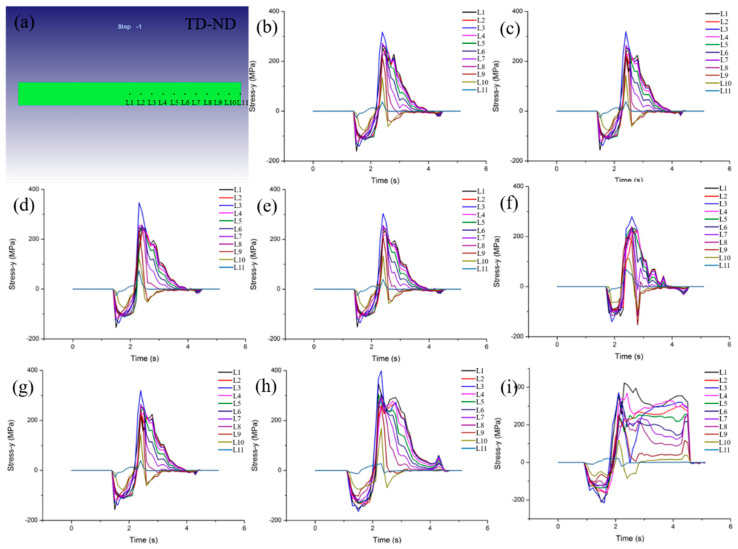
Distribution of characteristic points on TD−ND plane (**a**), the additive stress changes along TD at different temperatures (**b**–**e**) 1300–1600 °C, and different reductions (**f**–**i**) 10–40%.

Based on the results in Figure 9, the central characteristic points of the TD−ND plane thickness were further selected to simulate and analyze the variation in the additive stress along the TD direction, and these feature points are located at the 11 equal points from the center of the ND−TD plane to the edge as shown in Figure 10a. The additive tensile stress distribution at different rolling temperatures were carried out, as shown in Figure 10b–e. When the rolling temperature is 1300 °C, the maximum tensile stress is 317 MPa. At 1400 °C, the maximum tensile stress is 319 MPa. While the rolling temperature rises to 1500 and 1600 °C, the maximum tensile stress is 327 and 303 MPa, respectively. These data are the stress thresholds for forming longitudinal cracks at corresponding rolling temperatures. If the yield strength of the rolled sample itself exceeds the threshold of longitudinal crack formation, longitudinal crack will not occur in the rolled sample, otherwise, longitudinal crack will be formed. Additionally, the maximum tensile stress of ND−TD plane is basically unchanged at the set temperature range (1300–1600 °C), which further indicates that rolling temperature has little effect on longitudinal cracking. Moreover, it is worth noting that all the maximum tensile stresses appear at the characteristic point L3 in the test temperature range. This reason is complicated and needs further research in the future.

Figure 10f–i shows the tensile stress distribution of ND−TD plane at 1400 °C and different rolling reduction. The reduction is 10%, and the maximum tensile stress in TD direction is 280 MPa. While the rolling reduction increases to 20% and 30%, the maximum tensile stress is 319 and 399 MPa, respectively. Similar to Figure 10b–e, the maximum tensile stress along the TD direction also appears at L3. This indicates that the stress thresholds for longitudinal crack formation are 280, 319 and 399 MPa, corresponding to 10%, 20% and 30% of the reduction, respectively. However, when the reduction is 40%, the tensile stress curve shows two peaks. The first peak was at L3 with a tensile stress of 371 MPa, and the second peak was at L1, with a tensile stress of 423 MPa. As the reduction increases, the tensile stress along the TD direction obviously increases, and longitudinal cracking is more likely to occur. Moreover, when the reduction reaches 40%, the longitudinal cracking of the rolled tungsten plate changes from one crack type to two crack types. Thus, in order to avoid the formation of longitudinal cracks, low temperature and low pressure rolling are selected as far as possible within the set rolling temperature (1300–1600 °C) and the amount of pressure (10–40%).

**Figure 11:**
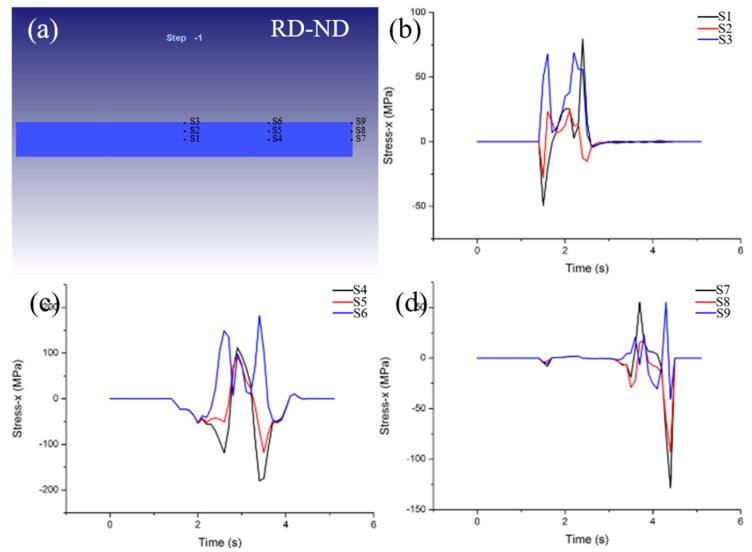
Distribution of characteristic points on RD−ND plane (**a**), the additive stress distribution along the RD direction of 9 selected feature points on the RD−ND plane at 1400 °C and 20% reduction (**b**–**d**).

Due to different parts of the constraints, uneven deformation occurs in the wide part of the rolling tungsten plat, resulting in the formation of additional tensile stress on the RD-ND plane. This can cause side crack formation. Figure 11 shows the stress distribution along the RD direction of nine selected feature points on the RD−ND plane at 1400 °C and 20% reduction. There is a significant difference in the maximum tensile stress along RD direction, the maximum tensile stress in the middle part is the largest and the minimum tensile stress in the tail part, so the side cracks preferentially appear in the middle part of the RD direction of the rolled sheet.

**Figure 12:**
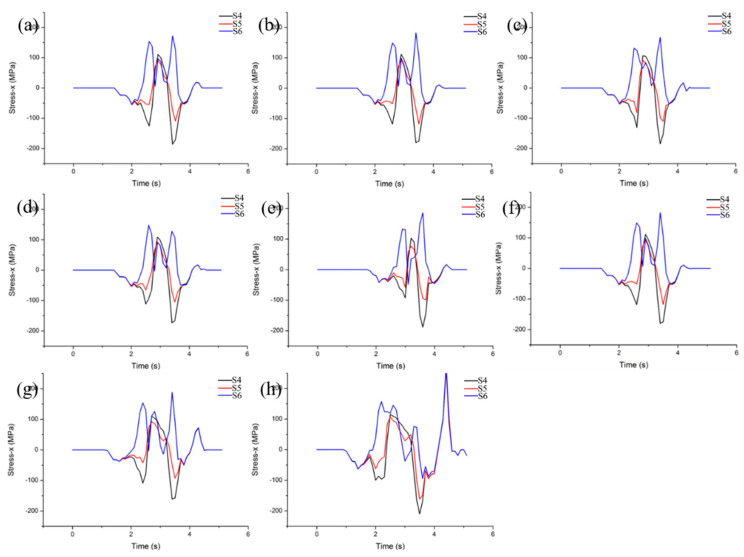
The additive tensile stress changes along TD on RD−ND plane at different temperatures (**a**–**d**) 1300–1600 °C, and different reductions (**e**–**h**) 10–40%.

Three characteristic points (S4, S5 and S6) with different thickness in the middle of RD−ND plane were selected to simulate and analyze the tensile stress under different rolling conditions, as shown in Figure 12. It can be seen from Figure 12a–d that there are three peaks of tensile stress along the RD direction at different rolling temperatures. Peaks 1 and 3 are larger and appear at characteristic point S6, while peak 2 is relatively smaller and appear at characteristic point S4. This indicates that the side cracks tend to form on the rolled surface. Moreover, the maximum tensile stress values in RD direction of S6 at different temperatures are 172, 182, 167 and 148 MPa, respectively. This shows that the maximum tensile stress along the RD direction decreases with increasing temperature after 1400 °C. Therefore, moderately increasing the rolling temperature can alleviate the formation of the side crack.

The reduction is 10%, and there are three stress extremes on the RD-ND plane, as shown in Figure 12e. Stress extreme values 1 and 3 appear at the characteristic point S6, which are 133 and 185 MPa, respectively. Since the extreme value 2 is small, it was not studied. Similarly, there are three extreme values of stress on the RD−ND plane with 20% reduction, and the stress extreme values 1 and 3 correspond to 149 and 182 MPa, respectively. However, the stress has four extreme peaks, and the extreme values 1, 3 and 4 all appear at the characteristic point S6, corresponding to 154, 180 and 72 MPa, respectively. Similar results were observed for 40% of the reduction. In particular, the fourth extreme peak was 277 MPa, which far exceeded the previous three peaks, as shown in Figure 12f–h. It shows that the stress distribution on the RD−ND plane changes from three peaks to four peaks with the increase in the reduction. Simultaneously, the stress of the first and fourth peaks increase rapidly, while the third peak decreases continuously. Further analysis shows that the first, second and third stress peaks appear in the biting stage, the rolling force transmitting to the center and the bottom stage, respectively, which is caused by the different transmission sequence of rolling force. However, the fourth extreme value appears after the end of rolling, which is caused by internal self-adjustment induced by uneven deformation. With the increase in the amount of reduction, the bite angle and rolling force increase, and the tensile stress at the first extreme value increases. In addition, the contact time between the rolled part and the roll is prolonged with the increase in the reduction, and the third extreme point stress is reduced. However, as the reduction continues to increase, the deformation inhomogeneity increases, thus resulting in the fourth stress value increasing. When the reduction is 10%, 20% and 30%, the side crack is controlled by the third stress extreme value, that is, the maximum deformation. The reduction is more than 40%, the side crack is controlled by the fourth stress extreme value, that is, the deformation uniformity. Thus, in order to suppress the side crack formation, the reduction should be controlled in the range of 20–30%.

Therefore, there are three kinds of hot rolling crack damage: transverse crack, longitudinal crack and side crack. By analyzing the values of the additive stress induced to three kinds of crack damage, it can be found that the longitudinal crack is the easiest to form and the main form of damage in the process of hot rolling of fine-grained pure tungsten plate. This is because the additional stress of longitudinal crack is the largest, exceeding 300 MPa (1300–1600 °C). Moreover, hot rolling crack is mainly the result of the competition between the additional stress caused by local inhomogeneous deformation and the mechanical properties (yield strength, elongation) of the material. Thus, the formation of hot rolling crack damage can be hindered by optimizing the preparation technology and microstructure to improve the high temperature strength, and selecting the appropriate reduction and temperature (20%, 1400 °C) to reduce the additive stress.

## 4. Conclusions

In this work, the fine-grained pure tungsten fabricated by sol drying reduction low-temperature sintering method was tested by hot compression tests using a Gleeble 3800 thermo mechanical simulator at deformation temperatures from 1273 K to 1473 K and strain rates from 0.001 s^−1^ to 1 s^−1^.

The constitutive equation and thermal deformation behavior were investigated. In addition, based on the constitutive relation of the ZA model, the hot rolling process was simulated by finite element method, and the effects of rolling temperature and reduction on temperature field, strain field, density field and rolling force have been discussed. The damage types and forming mechanism of hot rolling were further analyzed, and the control measures have been proposed. The pure W showed excellent superfine microstructure with an average grain size of only 5.22 μm. The flow stress increased with increased strain rates and decreased with increased temperatures, but there was no peak flow stress, which indicates that the flow behavior of fine-grained pure W at elevated temperatures shows typical dynamic recovery characteristics. The constitutive equation of as-sintered pure W for hot deformation has been described as: $\sigma =121.4+187.0exp\left(-0.094T+0.1T\ln\dot{\varepsilon }\right)+418.59\varepsilon^{0.477}$. Additionally, in the set rolling conditions (1300–1600 °C, 10–40%), compared with the rolling temperature, the reduction had a greater impact on the rolling temperature field, strain field, density field, rolling force and its distribution. Moreover, there were transverse cracks, longitudinal cracks and side cracks in the rolling process, which were caused by the additive stress in the ND direction and TD direction on the ND−TD plane, and RD direction on the RD−ND plane, induced by local inhomogeneous deformation, exceeding the yield strength of the material itself. Furthermore, the longitudinal crack was the easiest to form as well as the main form of damage, which can be hindered by improving the mechanical properties at high temperature and selecting the appropriate rolling technology (20% and 1400 °C).

## Figures and Tables

**Table 1:** Material parameters of fine-grained pure tungsten materials.

Density (g/cm^3^)	Young Modulus (GPa)	Poisson’s Ratio	Liner Expansion Coefficient (K^−1^)	Specific Heat Capacity (J/g/K)
18.72 [37]	354–400 [40]	0.28 [41]	5.6 × 10^−6^	0.125

**Table 2:** The fitting results of each parameter of the constitutive equation in ZA model.

Model	$\sigma_{0}$	$c_{1}$	$c_{3}$	$c_{4}$	$c_{5}$	n
ZA	121.4	187.0	9.4×10^−2^	0.10	418.59	0.477

## Data Availability

The data presented in this study are available on request from the corresponding author.
